# The effect of emotional labor and impression management on burnout: Example of Family Physicians

**DOI:** 10.12669/pjms.35.3.4

**Published:** 2019

**Authors:** Aysegul Ozer Yigit, Ferda Alper Ay

**Affiliations:** 1 *Aysegul Ozer Yigit, Health Care Management Graduate Student, Institute of Health Sciences, Sivas Cumhuriyet University, Cumhuriyet, Turkey*; 2 *Ferda Alper Ay, Department of Health Care Managment, Faculty of Health Sciences, Sivas Cumhuriyet University, Cumhuriyet, Turkey*

**Keywords:** Emotional Labor, Burnout, Impression Management, Family physicinas

## Abstract

**Objective:**

Although burnout is seen as a personal problem, it has a negative effect on effectiveness of the organization. The positive interaction of the physicians with the patients and their relatives increases the service quality. In this study, it was aimed to determine the effects of the family physicians’ use of emotional labor and impression management on burnout.

**Methods:**

A total of 82 questionnaires were distributed to all family physicians who work in the family health centers of the Public Health Directorate in Sivas province. The data were analyzed using descriptive statistics and multiple regression analysis.

**Results:**

According to the findings, the level of burnout of family physicians was low and there was a significant relationship between impression management tactics and emotional labor behavior. Impression management and emotional labor behaviors had no significant effect on burnout.

**Conclusion:**

Use of physicians’ emotional labor and impression management tactics, do not have an impact on their burnout levels and it helps improve physician-patient interaction.

## INTRODUCTION

At the end of 2010 in Turkey, a process of fundamental changes in primary health care services and physicians completed the tasks and working conditions has been redefined. According to the Family Medical Practice Regulation, family physicians are obliged to provide preventive health services for persons and primary care, diagnosis and rehabilitative health services in a certain place continuously and comprehensively; family physicians are doctors who provide mobile services when necessary”.^1^ Due to the nature of the profession of physicians and the fact that they have to communicate face to face with their patients, physical and mental labor as well as use intensive emotional labor are inevitable.^2^

When the literature was examined it was first used by Hochschild in 1983 emotional labor concept. According to Hochschild^3^, emotional labor is the process of showing or repressing feelings to show a facial expression that will create an appropriate situation in the minds of others as well as mental and physical work.^3^ Emotional labor has some benefits; helping to display behaviors appropriate to the employees’ code of conduct, helps the employee to develop his / her ability to express himself^4^, it helps to increase employee wages in performance based businesses, and, to prevent the employee from encountering negative behaviors and situations due to behaving in accordance with the rules determined by the institution and to protect the physical and mental health of the individual.^5^ On the other hand, in the working life, individuals have to manage their emotions in the direction of institutional expectations; this situation causes anxiety, alienation to their own emotions, emotional contradiction, role conflict.^6^ Emotional labor can also lead to emotional mismatch and job dissatisfaction.^7^

Individuals are trying to create the impression they want on their colleagues in the organization as well as on the people they meet in private life. In this respect, they try to control the way that individuals perceive others, to gain power, or to increase their power.^8^ Individuals, as a result of this information, are in an effort to influence and direct others’ perceptions and behaviors.^9^ Even if people are not aware of their impressions, impression management is emerging as a strategic behavior.^10^ Individuals use tactics of defensive (like innocence, finding an excuse) or aggressive impression management (like ingratiation, personal advertising). Thus, it can manage the image of other individuals who, while trying to create a positive image of themselves on others, may actually affect their own image.^11^

Burnout, as a concept, was first mentioned in an article by Freudenberger who described burnout as an occupational hazard.^12^ There are many personal and organizational reasons for burnout, the main reason being the excessive workload.^13^ It is known that there is a relationship between burnout syndrome and working conditions and environmental conditions. As the most important factors causing exhaustion in physicians; work load, daily working time, the number of patients and the number of seizures, sleep irregularities indicate working conditions.^14^

Meydan^15^ investigated the relationship between burnout and impression management dimensions. Meydan^15^ stated that individuals who experience burnout and who are trying to get rid of the psychological health problems they have created can use different impression tactics to manage their relationships within the organization. In the researchs, it was determined that the level of job satisfaction of the family physicians was moderate.^16^ On the other hand, the workload perception levels of the health workers working in the Public Hospital were examined and the health workers’ scores regarding the intention to pursue the same job were the lowest.^17^ Bartram et al.^18^ found that emotional labor was positively related to burnout and intent to leave work, and that burnout mediated the relationship between emotional labor and intent to leave work.

It is necessary for all employees and especially physicians in the health sector to behave in a gul-faced manner towards their patients and their relatives, give importance to their profession and to do business lovingly. If health workers show positive attitudes and behaviors, they will help patients and their relatives to show positive attitudes. For this reason, mutual positive interaction is beneficial for both service providers and for service recipients and increases service quality. In this study, we examined the effects of family physicians’ emotional labor and impression management use on burnout. In this study, in the presentation of health services of physicians, through emotional labor and use of impression management tactics, it helps physician-patient interaction. In addition, this study will help to expand the theoretical basis of health research.

From this point on, it was thought that emotional labor and impression management tactics would have a positive effect on burnout, and the hypotheses of the study are as follows.

### Hypotheses

H1: Emotional labor behaviors have a positive and significant effect on burnout.

H2: Impression management tactics have a positive and significant effect on burnout.

## METHODS

The universe of the research is based on 22 “Family Health Centers” affiliated with the “Sivas Provincial Public Health Directorate” there are totally 82 family physicians working (Alibaba, Aydogan, Carsibasi, Cayyurt, Demircilerardi, 4 September, Emek, Fatih, Gokmedrese, Gultepe, Kardesler, Karsiyaka, Guide, Kizilirmak, Mediko, M. Akif Ersoy, Architect Sinan, Orhangazi, Sheikhmashil, TOKI, Tuzlugol, Yunusemre). The sample of the study was composed of 80 family physicians who volunteered to participate in the research. This ratio constitutes approximately 98% (0,975) of the universe. Before starting the research, Cumhuriyet University was selected from the Ethical Committee for Non-Interventional Clinical Investigations (No. 2016-04 / 14) and from Sivas Public Health Directorate (No. 04/1/16, No. 73192166 / 044- E.764) approval has been obtained.

In the study, data were collected by questionnaire method. The questionnaire consists of four parts and 81 questions. In the first part, there are 5 questions about the demographic characteristics of family physicians. In the second part, 19 expressions are used to determine emotional labor behaviors. The “Emotional Labor Behavior Scale” was used in the study by applying the results of “Grandey^19^, Brotheridge and Lee^20^ “ and adapted to Turkish by Unler Oz.^21^ Cronbach alpha coefficients of the scale sizes found as a result of the scale adaptation study; role playing = 0.78, suppression = 0.77, in-depth behavior = 0.68.^22^

In the third phase, Impression management tactics scale was used to determine the impression management tactics of family physicians. This scale was developed by Demir^9^ on the basis of literature and construction validity was made. There are twelve dimensions on the scale of Impression management tactics consisting of 40 questions. The alpha internal consistency coefficients calculated for the reliability of the subscales of the scale range from 0, 76 to 0.55.

In the fourth chapter, a burnout scale consisting of 22 items and three sub-dimensions developed by Maslach and Jackson^23^ was used to measure burnout levels. The scale was translated into Turkish by Ergin^24^ and validated.^25^ The questionnaire form was applied between 1st April 2016 and 30 June 2016. In the analysis of the data, the relationship between the variables was examined by correlation analysis and regression analysis was used to test the hypotheses.

## RESULTS

When the characteristics of family physicians participating in the survey were examined, it was found that 60% of the participants were between the ages of 40-49, 63.8% were male, 78.8% were married, 30% had experience between 11-15 years and 100% ’s monthly income is 4001 TL and above (Table-I).

**Table-I:** Demographic characteristics of family physicians involved in the survey.

Age	N	%	Experience	N	%
30-39	23	28,8	1-5 years	4	5,0
40-49	48	60,0	6-10 years	10	12,5
50 and over	9	11,3	11-15 years	24	30,0
Cinsiyet			16 and over	42	52,5
Female	29	36,3	Income status		
Male	51	63,8	4001 and over	80	100,0
***Marital status***					
Married	63	78,8			
Single	17	21,3			

Total	80	100	Total	80	100

When relations between variables were examined, there was a positive correlation between emotional labor behavior and impression management tactics (r = 0.306). Positive relations between emotional labor and burnout and impression management and burnout are not significant. In addition, participants’ emotional labor behavior levels (3,42), impression management tactics (2,97), and burnout level (2,45) were generally determined (burnout level was found to below) (Table-II).

**Table-II:** Descriptive Statistics.

Variables	Average	Std deviation	Correlations		

			1	2	3
1. Impression management	2,97	0,48	(0,879)		
2. Emotional labor	3,42	0,50	0.306(**)	(0,671)	
3. Burnout	2,45	0,50	0.120	0.137	(0,832)

** p <0,01 significance level relationship was significant. Values in parentheses indicate reliability values.

As can be seen from Table-III, the multiple regression model between impression management tactics and emotional labor behavior and burnout was not significant (F =0,010, p= 0.369). H1 and H2 hypotheses was therefore rejected.

**Table-III:** The impact of emotional labor behavior and impression management tactics on burnout.

Independent Variables	Dependent Variable	Model Summary		ANOVA		Regression coefficients			Hypothesis	Result

		R	R^2^	F	P	Beta	t	p		
Impression Management	Burnout	0,160	0,026	1,010	0,369	,086	,731	,467	H4	Rejection
Emotional Labor						,111	,937	,352	H5	Rejection

*P<0,05, ** P<0,01 Durbin-Watson=1,344

## DISCUSSION

The result of our study is different and more positive than the other studies in the sample of health workers. In the literature, emotional labor behaviors, and impression management tactics have been found to increase burnout in health workers in general, but not enough studies have been done in the sample of family physicians.

At the end of the research, family physicians’ burnout levels were low. Similarly, Serik et al.^26^ found that burnout levels were generally low in the study conducted with 157 family physicians working in family health centers in Sakarya province.

In our study, it was concluded that emotional labor and impression management tactics had no effect on the burnout of physicians. Similar to the results of the study, in the studies carried out by Yurur and Unlu^4^ tourism enterprises, it was determined that superficial behavior and in-depth behavior from emotional labora dimensions had no significant effect on emotional exhaustion. This result supports our research results.

Unlike our findings, a significant relationship was found between impression management and burnout in a survey conducted in the banking sector.^15^ Esen et al.^14^ High job satisfaction and personal success and low emotional exhaustion were observed in the socially active individuals in family medicine residents. This result is similar in terms of burnout levels of family physicians in our study.

In another study conducted with doctors, superficial behavior was found to increase the level of emotional burnout and depersonalization of individuals, but no relation between in-depth behavior and emotional burnout was found.^2^

Unlike other physicians, this result may be due to the fact that the working environment of the family physicians is better, patients do not come to the family doctors with serious diseases because they offer primary health care services. It may also be due to the fact that the family physicians are less exhausted than the other physicians and health workers.

## CONCLUSION

The results of the study show the importance of increasing the quality of health care services through the use of tactics of emotional labor and impression management to help physicians understand patients. The physician tries to understand the patient, for the patient to choose the same physician again and to choose the same health institution, demonstration of these behaviors may be recommended to physicians.The use of emotional labor behaviors and impression management tactics for family physicians does not lead to burnout.
